# Serum Protein Pattern Could Predict the Therapeutic Effect of First-Line Pemetrexed/Cisplatin Chemotherapy in Patients With Lung Adenocarcinoma

**DOI:** 10.14740/wjon901w

**Published:** 2015-02-14

**Authors:** Hong Shen, Xue-Feng Fang, Ying Yuan, Jiao Yang, Shu Zheng

**Affiliations:** a Department of Medical Oncology, 2nd Hospital of Zhejiang University College of Medicine, China; b Key Laboratory of Cancer Prevention and Intervention of Ministry of Education, 2nd Hospital of Zhejiang University College of Medicine, China

**Keywords:** Surface-enhanced laser desorption/ionization time-of-flight mass spectrometry, Artificial neural networks, Non-small cell lung cancer, Chemotherapy, Protein pattern, Adenocarcinoma

## Abstract

**Background:**

In patients with advanced non-squamous non-small cell lung cancer (NSCLC), a pemetrexed/cisplatin (PP) regimen is considered as one of the preferred first-line treatments. However, only about half of the treated patients respond, and there is no clinically useful marker that can predict the response to the regimen.

**Methods:**

We established a potential pattern for the prediction of efficacy of first-line PP chemotherapy in patients with lung adenocarcinoma, by using artificial neural networks (ANNs) analysis of surface-enhanced laser desorption/ionization time-of-flight mass spectrometry (SELDI-TOF-MS) in this preliminary study.

**Results:**

The samples were randomly divided into training set and test set. From the test set, through cross-validation, the established protein pattern for PP separated the responders from the non-responders with a sensitivity of 95.8% and a specificity of 90.0%.

**Conclusion:**

It could be helpful for oncologists to select patients who could benefit from PP chemotherapy.

## Introduction

Lung cancer is one of the most prevalent cancers and the leading cause of cancer death worldwide. Non-small cell lung cancer (NSCLC) represents 85% of all lung cancers. The 5-year survival of patients with metastatic NSCLC is less than 10% [1, 2]. Platinum-based doublet chemotherapy is the current standard of care for chemotherapy naive patients with preserved functional status. Patients treated with platinum-based regimens have a mean survival of 8 - 10 months.

Pemetrexed is a multi-targeted inhibitor of three key enzymes in the folate metabolic pathway: thymidylate synthase (TS), dihydrofolate reductase (DHFR), and glycinamide ribonucleotide formyl transferase (GARFT) [3, 4]. In 2008, Scagliotti et al [5] first reported a large phase III clinical trial to compare first-line pemetrexed/cisplatin (PP) to gemcitabine/cisplatin (GP) and found that pemetrexed significantly improved OS in non-squamous NSCLC patients. Based on this study, pemetrexed has been designated as the first-line treatment for patients with advanced non-squamous NSCLC. However, this new combination remains inactive in about half of the patients, and resistance to treatment appears in almost all patients who were initially responders. With the development of individualized therapies for various types of malignant tumors, the establishment of a chemotherapeutic regimen based on sensitivity-associated markers for NSCLC patients is urgently needed.

Surface-enhanced laser desorption/ionization time-of-flight mass spectrometry (SELDI-TOF-MS), a recently developed technology based on capturing peptides and/or proteins by chemically modified surfaces, is particularly powerful for analyzing complex biological samples. Combined with bioinformatics, SELDI-TOF-MS has revealed several biomarkers with a high sensitivity and specificity for the diagnosis of cancer [6-10]. Moreover, when compared with a two-dimensional gel electrophoresis (2D-GE), SELDI-TOF-MS is cost-effective and has a high throughput. The aim of our present study was to identify potential biomarkers and/or protein patterns to distinguish responders from non-responders to PP chemotherapy in patients with lung adenocarcinoma using SELDI-TOF-MS and artificial neural networks (ANNs).

## Materials and Methods

### Collection of patients and samples

From August 2009 to July 2010, 92 patients with metastatic lung adenocarcinoma at the Department of Medical Oncology, 2nd Hospital of Zhejiang University College of Medicine were enrolled in this study. All of the samples were obtained from the serum bank of the Cancer Institute of Zhejiang University. All participating patients were informed of the purpose of the study and signed a written consent before sample and clinical information were collected. The study protocol has been approved by the institute’s committee on human research.

The eligibility criteria for inclusion were as follows: chemotherapy naive, histologically proven adenocarcinoma of the lung, with at least one measurable lesion, age ranging from 18 to 75 years old, and WHO performance status of 1 or less.

All blood samples were collected before chemotherapy and in the morning before food intake. They were centrifuged at 1,000 rpm for 2 min and the serum were transferred to other tubes, and stored at -80 °C until use.

### Chemotherapy

Ninety-two patients were treated with a PP regimen as first-line chemotherapy. Patients received 500 mg/m^2^ pemetrexed on day 1 plus 25 mg/m^2^ cisplatin on day 1 to day 3. Chemotherapy was repeated every 3 weeks for a maximum of six cycles. Patients received dexamethasone prophylaxis of 4 mg orally twice per day on the day before, the day of, and the day after each day-1 treatment. Patients received oral folic acid (400 μg) daily and a vitamin B12 injection (1,000 μg) every 9 weeks, beginning 1 - 2 weeks before the first dose and continuing until 3 weeks after the last dose of study treatment.

The efficacy of the chemotherapy was evaluated according to the RECIST criteria for solid tumors [11]. The size of the lesions was determined from dimensional measurements (the longest diameter) using computed tomography scanning. Patients were evaluated for response after every two cycles of chemotherapy. The best observed response was then used to classify patients into two groups. Complete response (CR) is defined as the disappearance of all target lesions and reduction of the short axes of target lymph nodes to < 10 mm. Partial response (PR) was defined as a decrease in the sum of the diameters of all target lesions by ≥ 30%. Progressive disease (PD) was defined as an increase of ≥ 20% (with at least a 5-mm increase) in the sum of target lesion diameters or the appearance of new lesions. If no response or progression of the disease occurred during the chemotherapy, then the therapeutic effect was judged to be stable disease (SD). The responders (R) were defined as patients with CR, PR or SD, while patients with PD were defined as non-responders (NR) in this study.

### SELDI-TOF-MS

The SELDI-TOF-MS technique was as follows. Serum samples in ice were thawed and then centrifuged at 3,000 rpm for 5 min at 4 °C. The supernatants were retained. Ten microliters of each sample were added into 90 μL of 5 g/L 3-((3-cholamidopropyl) dimethylammonio)-1-propanesulfonate (CHAPS; Sigma, St. Louis, MO, USA), pH 7.4 and the mixtures were vortex for 10 min. The diluted samples were then added to 100 μL Cibacron Blue 3GA (Sigma), which were previously equilibrated three times with 5 g/L CHAPS, in 96-well cell culture plates and then agitated on a platform shaker at 4 °C for 60 min. After centrifugation at 1,000 rpm, 50 μL supernatants were removed, then further diluted by 150 μL of 20 mmol/L HEPES, pH 7.4 and applied to each well of a bioprocessor (Ciphergen Biosystems, Fremont, CA, USA) containing hydrophobic surface (H4) chips, previously activated by 20 mmol/L HEPES. The bioprocessor was then sealed and agitated on a platform shaker at 4 °C for another 60 min. The excess serum mixtures were discarded, and the chips were washed three times by shaking gently on a platform shaker at a speed of 700 rpm for 5 min in 200 μL of 20 mmol/L HEPES, pH 7.4. The chips were air dried, and crystallized by the addition of acyano-4-hydroxycinnamic acid (CHCA; Ciphergen Biosystems).

Chips were finally assayed on the Protein Biological System II and mass spectrometer reader (Ciphergen Biosystems). Data were collected by averaging 65 laser shots with an intensity of 165, a detector sensitivity of 7, a highest mass of 60,000 m/z, and an optimized range between 2,000 and 30,000 m/z. Mass accuracy was calibrated to less than 0.1% using the all-in-1 peptide molecular mass standard (Ciphergen Biosystems). The spectra of all samples were normalized to the total ion current of mass to charge ratios (m/z) from 2,000 to 30,000. Noise was filtered from the spectra and peaks were detected using an automatic peak detection pass. A second pass peak selection (signal-to-noise ratio > 2, within a 0.3% mass window) was employed to complete peak clusters. Estimated peaks were added. ProteinChip Software 3.2 and Biomarker Wizard 3.1 (Ciphergen) were used to perform all these data collection steps.

The ANNs are able to recognize complex patterns in measured input variables that are not apparent to other forms of analyses. In the present study, it was employed to analyze the collected protein mass-dependent velocity (m/z) peaks. After processing the incoming data by several steps of transformation, the networks could produce an output, indicating a specific category within a given classification. All of the calculations were made with Statistica 6.0 (StatSoft, Inc., Tulsa, OK, USA) software package.

## Results

### Efficacy of chemotherapy

In 92 cases treated with PP regimen, 71 patients (71/92, 77.2%) were determined to be responders whereas the other 21 patients (21/92, 22.8%) were non-responders.

### Results of SELDI-TOF-MS and ANNs

After filtering noise using the Ciphergen ProteinChip Software, a total of 370 peaks from specimens from patients with metastatic lung adenocarcinoma treated with PP regimen, were identified to be potential biomarkers that could predict treatment efficacy. All these peaks corresponded to the m/z of serum proteins of 2,000 to 30,000. Those with the m/z of less than 2,000 were mainly ion noise from the matrix and therefore excluded.

**Figure 1:**
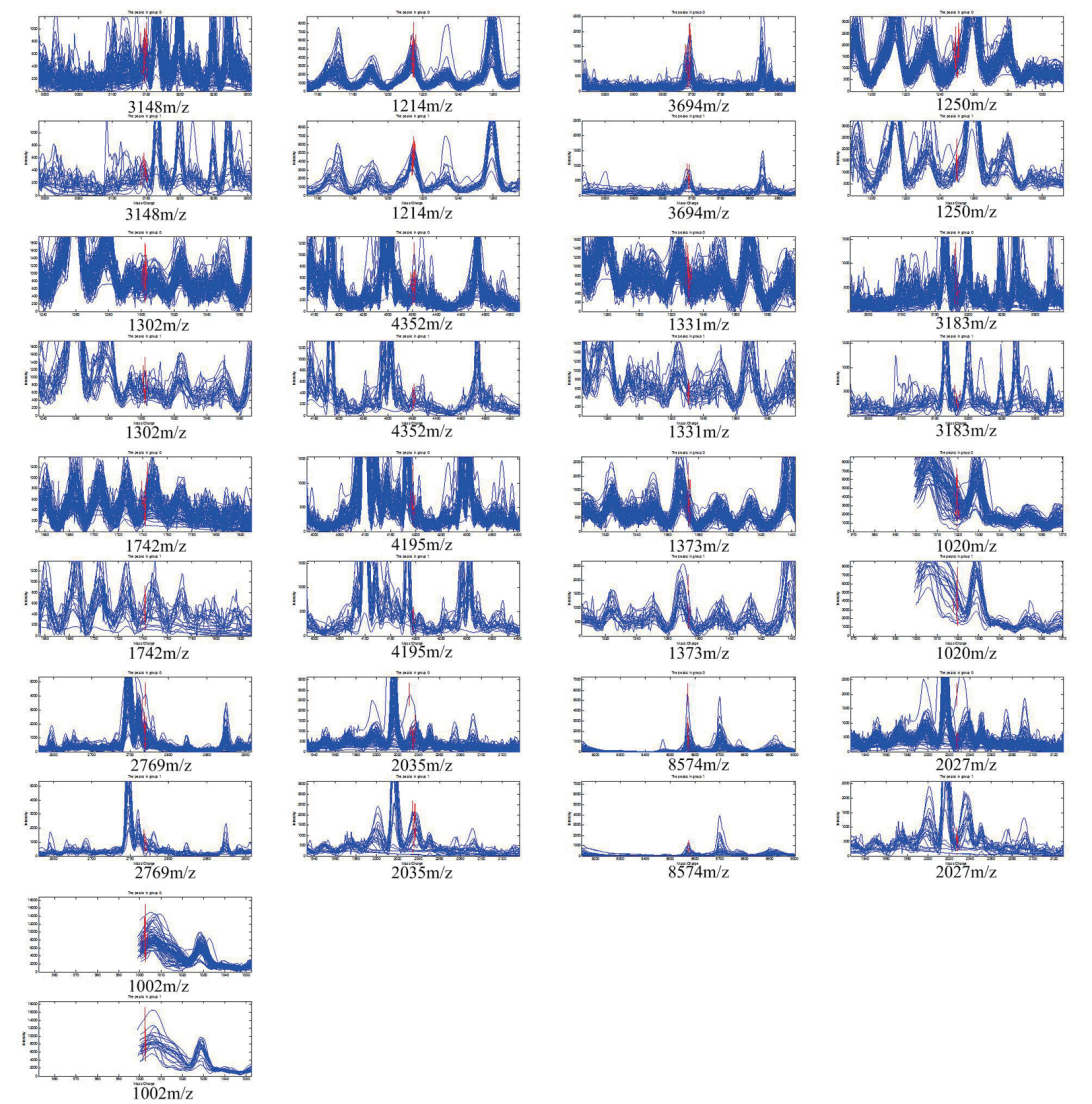
The 17 protein peaks with the highest area under the curve detected in the patients treated with pemetrexed/cisplatin regimen. The peaks of 3,148 m/z, 3,694 m/z, 1,250 m/z, 1,302 m/z, 4,352 m/z, 1,331 m/z, 3,183 m/z, 1,742 m/z, 4,195 m/z, 1,373 m/z, 2,769 m/z, and 8,574 m/z had higher expression in responders. The peak of 1,214 m/z, 1,020 m/z, 2,035 m/z, 2,027 m/z, and 1,002 m/z had lower expression in responders.

The 370 qualified peaks detected from the two groups (responders vs. non-responders) of patients treated with the PP regimen were ranked by means of a receiver operating characteristic (ROC) curve. Using the stepwise approach, final 17 peaks with the highest area under the curve were selected as potential biomarkers. The m/z of the 17 candidates were: 3,148 m/z, 1,214 m/z, 3,694 m/z, 1,250 m/z, 1,302 m/z, 4,352 m/z, 1,331 m/z, 3,183 m/z, 1,742 m/z, 4,195 m/z, 1,373 m/z, 1,020 m/z, 2,769 m/z, 2,035 m/z, 8,574 m/z, 2,027 m/z, and 1,002 m/z. The peaks corresponding to 3,148 m/z, 3,694 m/z, 1,250 m/z, 1,302 m/z, 4,352 m/z, 1,331 m/z, 3,183 m/z, 1,742 m/z, 4,195 m/z, 1,373 m/z, 2,769 m/z and 8,574 m/z were much more highly expressed in the responders than in non-responders, while the other peaks, 1,214 m/z, 1,020 m/z, 2,035 m/z, 2,027 m/z and 1,002 m/z were lower in the responders (Fig. 1). The P values obtained from the *t* tests and the area under the ROC curve indicated statistical significance for all 17 peaks (Table 1). With these 17 peaks, a protein pattern for the prediction of PP regimen efficiency was established.

**Table 1:** The Statistics of the 17 Protein Peaks Detected in the Patients Treated With Pemetrexed/Cisplatin Regimen, Mean and Standard Deviation (SD)

m/z	P value	R (mean ± SD)	NR (mean ± SD)
3,148	0.000109	521.81 ± 175.4	355.91 ± 103.57
1,214	0.002012	3,926.37 ± 969.80	4,647.60 ± 841.31
3,694	0.003286	689.87 ± 363.65	453.64 ± 206.30
1,250	0.004046	1,399.20 ± 367.01	1,157.82 ± 306.77
1,302	0.007588	838.06 ± 246.64	691.54 ± 236.32
4,352	0.007801	344.69 ± 144.04	260.71 ± 99.07
1,331	0.008019	645.74 ± 225.81	512.14 ± 108.18
3,183	0.008472	408.48 ± 229.04	267.72 ± 124.61
1,742	0.010242	489.42 ± 182.47	375.91 ± 172.00
4,195	0.011097	387.48 ± 164.91	278.95 ± 119.33
1,373	0.013693	855.47 ± 293.54	719.61 ± 318.68
1,020	0.018593	3,264.40 ± 1,213.55	4,066.30 ± 1,458.65
2,769	0.023216	1,066.17 ± 709.69	692.81 ± 379.49
2,035	0.024369	669.12 ± 384.69	1,026.08 ± 673.00
8,574	0.0268	757.66 ± 808.53	427.4741 ± 361.53
2,027	0.027472	378.24 ± 244.43	466.69 ± 214.87
1,002	0.049661	7,287.71 ± 1,891.80	8,121.41 ± 2,056.97

The samples were randomly divided into training set and test set. From the test set, through cross-validation, the established protein pattern for PP separated the responders from the non-responders with a sensitivity of 95.8% and a specificity of 90.0%.

## Discussion

Lung cancer remains the leading cause of death worldwide. Platinum-based doublets are used as standard first-line chemotherapy in NSCLC patients, with an objective response rate of 30-40%, a median survival time of 8 - 10 months, and a 1-year survival rate of 30-40% [12, 13]. However, even patients with the same demographic and clinical characteristics usually display different responses and varied prognoses. Thus, the concept of individualized chemotherapy becomes more intriguing, and one of the remaining challenges is the development of predictive markers to individualize and optimize patient therapies. Given that the diagnosis of advanced cancers is mainly based on a small needle biopsy sample, using tumor tissue to acquire further predictive information may be difficult. Thus, identifying biomarkers from peripheral venous blood as predictive markers becomes much more appealing, especially in the advanced cancer setting.

There have been many attempts to determine predictive factors for response in NSCLC. Boukovinas et al [14] reported the mRNA expression of BRCA1, RRM1 and RRM2 is potentially a useful tool for first-line gemcitabine plus docetaxel for NSCLC patients. Park et al [15] demonstrated that lung cancer patients with EGFR mutations had longer PFS with taxane than gemcitabine when receiving a platinum-based doublet regimen. However, the predictive efficacy of single markers remains insufficient; therefore, their use in routine clinical practice has been limited. Furthermore, many study indicated that the measurement of multiple rather than single marker resulted in a more accurate assessment of drug response. Up to now, there are limited reports concerning the serum protein levels or patterns for predicting a response to chemotherapy in NSCLC patients. Taguchi et al [16] reported a matrix-assisted laser desorption ionization (MALDI) mass spectrometry (MS) algorithm that could classify NSCLC patients with good or poor outcomes after treatment with EGFR TKIs. Han et al [17] demonstrated serum biomarkers identified by SELDI-TOF MS that could predict chemotherapy resistance in patients with advanced NSCLC.

SELDI-TOF-MS protein chip array technology was used to find several peaks that were different in the responders and non-responders. ANNs were then employed to analyze these peaks, and finally established a protein pattern predicting the efficacy of a PP regimen. This predicting pattern was effective for distinguishing responders from non-responders in the test set. The sensitivity and specificity of the protein pattern were 95.8% and 90.0%, respectively. Therefore, it is particularly important to further identify these proteins or peptides. If their molecular nature could be clarified, their application for the prediction of efficacy of chemotherapy would be greatly expanded.

This study provides new insights into the treatment of metastatic NSCLC. A major application would be to use the serum protein expression pattern in the first-line metastatic setting as a tool to assist oncologists in selecting suitable adenocarcinoma patients who could benefit from chemotherapy. By using the predicting pattern, we were able to detect almost all the responders from our cohort. The advantage of our mono-center study is that the genomic and clinical data quality was homogeneous. However, as the results were based on a relatively small sample size, it is essential to validate and improve the pattern in a larger independent cohort of patients.

In conclusion, SELDI-TOF-MS, the proteomic technology, in combination with the bioinformatics tool, ANNs, can facilitate the discovery of new biomarkers. The data from the study contribute significant information on the predictive value of serum protein patterns and may prove to be a useful tool working towards individualizing NSCLC treatment strategies. High sensitivity and specificity of patterns can be achieved in this manner.
